# Evolving Trends in the Epidemiology, Resource Utilization, and Outcomes of Pregnancy-Associated Severe Sepsis: A Population-Based Cohort Study

**DOI:** 10.14740/jocmr2118w

**Published:** 2015-04-08

**Authors:** Lavi Oud, Phillip Watkins

**Affiliations:** a Division of Pulmonary and Critical Care Medicine, Department of Internal Medicine, Texas Tech University Health Sciences Center at the Permian Basin, 701 W. 5th St., Odessa, TX 79763, USA; b Clinical Research Institute, Texas Tech University HSC, 3601 4th Street, MS6238, Lubbock, TX 79430, USA

**Keywords:** Intensive care unit, Mortality, Pregnancy, Resource utilization, Severe sepsis

## Abstract

**Background:**

Infections are a well-known complication of pregnancy. However, pregnancy-associated severe sepsis (PASS) has not been as well-characterized, with limited population-level data reported to date. We performed a population-based study of the evolving patterns of the epidemiology, clinical characteristics, resource utilization, and outcomes of PASS in Texas over the past decade.

**Methods:**

The Texas Inpatient Public Use Data File was used to identify pregnancy-associated hospitalizations and PASS hospitalizations for the years 2001 - 2010. The Texas Center for Health Statistics reports of live births, abortions and fetal deaths, and a previously reported population-based, age-specific linkage study on miscarriage were used to derive the annual total estimated pregnancies (TEPs). The incidence, demographics, clinical characteristics, resource utilization and outcomes of PASS were examined. Logistic regression modeling was used to explore the predictors of PASS and its associated mortality.

**Results:**

There were 4,060,201 pregnancy-associated hospitalizations and 1,007 PASS hospitalizations during study period. The incidence of PASS was increased by 236% over the past decade, rising from 11 to 26 hospitalizations per 100,000 TEPs. The key changes between 2001 - 2002 and 2009 - 2010 within PASS hospitalizations included: admission to ICU 78% vs. 90% (P = 0.002); development of ≥ 3 organ failures 9% vs. 35% (P < 0.0001); and inflation-adjusted median hospital charges (2,010 dollars) $64,034 vs. $89,895 (P = 0.0141). Hospital mortality (11%) remained unchanged during study period. Chronic liver disease (adjusted odds ratio (aOR) 41.4) and congestive heart failure (CHF) (aOR 20.5) were associated with the highest risk of PASS, in addition to black race, poverty, drug abuse, and lack of health insurance. The highest risk of death was among women with HIV infection (aOR 45.5), need for mechanical ventilation (aOR 4.5), drug abuse (aOR 3.0), and lacking health insurance (aOR 2.9).

**Conclusions:**

The incidence, severity, and fiscal burden of PASS rose substantially over the past decade. Case fatality was lower than that for severe sepsis in the general population. Chronic liver disease and CHF pose especially high risk of PASS. Pregnant women with history of drug abuse and lacking health insurance are at high risk of both developing and dying with PASS, requiring extra vigilance for early diagnosis and targeted intervention.

## Introduction

The incidence of severe sepsis in the general population is rapidly increasing and is associated with high morbidity and mortality [1-3]. The global burden of sepsis has been estimated by Adhikari and colleagues to range from 15 to 19 million cases per year [4], and a recent report estimated that septicemia is the most expensive condition among hospitalized patients in the United States [5].

Despite its increasing incidence and the personal and economic burdens, major strides were made over the past decade in improving the outlook for patients with severe sepsis. Several landmark studies have documented improved patient outcomes with timely targeted circulatory resuscitation [6] and administration of appropriate antibiotics [7] in severely septic patients. Recent reports have documented that incorporating guideline-based bundled care [8] into clinical practice was associated with reduced mortality [9]. A substantial part of the aforementioned progress in our understanding of the epidemiology of severe sepsis and improvements in its management stems from the standardization of case definitions of sepsis, severe sepsis, and septic shock [10, 11].

However, the aforementioned strides have not been fully realized in the obstetric population. Pregnancy is associated with increased risk of infection, related to various pregnancy-related mechanical, physiological [12], and immunity-related [13] changes. Although there has been tremendous progress in reducing maternal morbidity and mortality related to pregnancy-associated infectious complications, the latter remain a major source of pregnancy-related mortality in both developing and developed countries worldwide, reported to be the third to fourth most common cause of maternal death [14]. A recent review conducted by the World Health Organization has estimated the global burden of maternal sepsis to be more than 6,900,000 cases per year [15].

Many investigators [14, 16-18] have noted that one of the more basic ongoing challenges to our understanding of the burden of pregnancy-associated sepsis and development of severe sepsis among infected patients is that clinical reports often employ imprecise and variable terminology, using (often interchangeably) terms such as septicemia, sepsis, puerperal infection, puerperal fever, or maternal sepsis, thus affecting both clinical practice and present knowledge about maternal sepsis and severe sepsis in the obstetric population. Despite the voluminous body of published research on pregnancy-associated infections and sepsis, our contemporary understanding about pregnancy-associated severe sepsis (PASS) remains sparse.

There are several explanations for this knowledge gap. These include the following limitations of available data: 1) published reports to date rarely focused explicitly and/or primarily on PASS; 2) when reported, studies commonly varied in their case definition of severe sepsis [19-23], often at variance with those used in the general population, limiting inference and comparison across studies or with the general population; 3) varying methodological approaches were used in studies of PASS to estimate its incidence, further limiting comparisons across studies; 4) sample size of reported PASS studies has often been small [19-21] and often reflected local rather than population-level data, further limiting inferences from provided data; and 5) reports on PASS focused at times on selected periods of pregnancy (i.e., delivery) [22, 24], affecting inference about the burden of PASS across the full spectrum of pregnancy.

The aims of the present study were to: 1) examine the contemporary patterns of the epidemiology, key clinical features, resource utilization, and outcomes of PASS across the full spectrum of pregnancy phases and its key outcomes, and 2) determine the risk factors for development of PASS and its associated mortality.

## Material and Methods

### Setting and data sources

We used the Texas Inpatient Public Use Data File (TIPUDF), a longitudinal data set maintained by the Texas Department of State Health Services [25] to perform a retrospective, population-based cohort study of PASS in the state. The data set includes detailed de-identified inpatient discharge data from all state-licensed hospitals, with the exception of those exempt by state statute from reporting to the Texas Health Care Information Collection. Exempt hospitals include 1) those that do not seek insurance payment or government reimbursement and 2) selected rural providers, based on bed number and local county population. The facilities included in the mandated report account for 93-97% of all hospital discharges. The TIPUDF data set includes demographic, clinical, resource utilization, and outcome information. The data set includes up to 25 discharge diagnoses, and up to 25 procedures, coded using the International Classification of Diseases, Ninth Revision, Clinical Modification (ICD-9-CM).

Data on the annual number of pregnancies, live births, abortions, fetal deaths, and their related demographic characteristics were obtained from the Vital Statistics Annual Reports, compiled by the Center for Health Statistics at the Texas Department of State Health Services [26].

US Census data [27] were used to derive information on data on the proportion of residents living below poverty line of the population residing at the zip code of women with pregnancy-associated hospitalizations. Because we used a publicly available, de-identified data set, this study was determined to be exempt from formal review by the Texas Tech Health Sciences Center Institutional Review Board.

### Study population

We used ICD-9-CM codes (Supplementary Table 1, www.jocmr.org) to identify Texas residents with pregnancy-related hospitalizations between 2001 and 2010. PASS was defined as a combination of pregnancy-related diagnosis and a primary or secondary diagnosis of severe sepsis. The case definition of severe sepsis was modeled on the coding system reported by Lagu et al [2]. Specifically, severe sepsis was defined as primary or secondary diagnosis codes of either: 1) an ICD-9-CM code for either septic shock (785.52) or systemic inflammatory response syndrome due to an infectious process with organ failure (995.92) and/or 2) ICD-9-CM codes for an infectious process (Supplementary Table 2, www.jocmr.org) with a diagnosis of at least one organ failure (Supplementary Table 3, www.jocmr.org). The severity of illness was assessed by the number of failing organs [1, 28].

### Data collection

We collected data on patients’ age, race (categorized as non-Hispanic black (black), non-Hispanic white (white), Hispanic, and other), health insurance (categorized as private, Medicaid, uninsured, and other), zip code at area of residence, chronic co-morbid conditions (based on the Deyo modification of the Charlson co-morbidity index [29]), obesity, smoking, drug and alcohol abuse, hospital’s teaching status, pregnancy-associated complications, high-risk factors and delivery-related procedures (Supplementary Table 4, www.jocmr.org), sites of infection (Supplementary Table 5, www.jocmr.org), reported microorganisms (Supplementary Table 6, www.jocmr.org), type and number of failing organs, admission to an ICU (defined as presence of an intensive care unit charge greater than $0), life support-related interventions (mechanical ventilation, central venous catheterization, and hemodialysis) (Supplementary Table 7, www.jocmr.org), teaching status of the hospital, total hospital charges, hospital length of stay, and disposition at the end of hospitalization. We categorized the type of pregnancy-associated hospitalizations into the following mutually exclusive, hierarchical groups, using pregnancy-associated ICD-9-CM codes: 1) fetal loss (pregnancies with abortive outcome, excluding induced abortion); 2) induced abortion; 3) delivery (based on the approach described by Kuklina et al [30]); 4) postpartum (hospitalizations with an ICD-9-CM code for puerperal complications, without pregnancy-related diagnosis codes of groups 1 - 3), and 5) antepartum (hospitalization with pregnancy-related diagnosis, but without pregnancy-related diagnosis codes of groups 1 - 4).

### Outcomes

The primary outcome was hospital mortality. Secondary outcomes included the number and type of failing organs, resource utilization and disposition among hospital survivors.

### Data analysis

In order to derive the incidence of PASS events across the full spectrum of pregnancy population at risk, we calculated the annual total estimated pregnancies (TEPs). TEP was a combination of the number of live births, fetal deaths (events reported by the state, occurring at ≥ 20 weeks of gestation), induced abortions, and estimates of the annual number of fetal losses (events occurring at < 20 weeks of gestation, including miscarriage, ectopic and molar pregnancies). The estimation of the annual number of fetal losses was based on the findings reported by Nybo Anderson et al [31]. This was a population-based linkage study of the association of maternal age with fetal loss, reporting rates of fetal loss for pregnancies intended to be carried to term, thus adjusting for overestimates resulting from fetal loss events prior to planned abortion. We used these rates with reported annual number of live births and fetal deaths to derive the estimated number of fetal losses and then TEP. Because TIPUDF provides discharge-level, rather than patient-level information, we reported PASS events as number of hospitalizations. We used direct standardization to calculate age-adjusted incidence rates of patients’ hospitalizations with a diagnosis of PASS per 100,000 TEPs. In addition, although the primary focus of our study has been to estimate the incidence of PASS across the full spectrum of pregnancy population, we have performed further subgroup analyses to allow better comparison with prior reports that focused on delivery or live birth hospitalizations, and to examine PASS events associated with unintended or induced termination of pregnancy. Due to the low number fetal loss- and abortion-related PASS events, these incidence estimates were based on the total PASS events in a given group over study period. Twenty-six PASS hospitalizations associated with fetal loss/induced abortion could not be adequately classified to only one group (that is, either fetal loss or induced abortion), because their only pregnancy-associated ICD-9-CM code was 639.XX (complications following abortion and ectopic and molar pregnancies). We re-calculated upper estimates of incidence and mortality rate (reported parenthetically) for both fetal loss and induced abortion among PASS hospitalizations, assuming alternately that the unclassified hospitalizations were only fetal loss- or only induced abortion-related. To assure consistency, we used the term fetal loss throughout the manuscript to denote the terms spontaneous abortion or miscarriage used in other reports.

We performed multiple sensitivity analyses to examine the robustness of our incidence estimates. Although TIPUDF is reported to include 93-97% of annual hospital discharges, we reanalyzed the annual incidence of PASS for the possibility that the dataset captures only 90% of all hospital discharges (that is, extrapolating the annual PASS incidence to 100% reporting), and that the incidence of PASS was 50% higher in non-reporting hospitals. In addition, due to the uncertainty about the accuracy of estimated fetal losses and resultant TEP, we reanalyzed the annual incidence of PASS, assuming that the rate of fetal loss among Texas residents is 100% higher than the 13.5% figure reported by Nybo Anderson et al [31]. This higher rate (27%) exceeds the upper estimated rate of fetal loss of 22% reported in a recent systematic review by Ammon Avalos and colleagues [32]. Because changes in frequency of reported organ failures over time may represent over-coding [2], we compared the rates of utilization of organ-specific life support-related interventions among severe sepsis hospitalizations with a specific organ failure (i.e., use of mechanical ventilation among hospitalizations with reported respiratory failure) at the start and end of study period.

The mortality associated with PASS was examined as both case fatality (defined as the number of PASS hospitalizations who died in the hospital divided by the total number of PASS hospitalizations for an examined group) and as mortality rate per 100,000 TEPs. Trends of the annual case fatality and mortality rates were examined using log-transformed regression analysis. We performed further subgroup analyses of mortality rates based on the type of pregnancy outcome (fetal loss and induced abortion) and that of delivery hospitalizations for further comparison within groups and with prior reports, using similar approach to that described for estimates of subgroup incidence.

We constructed multiple logistic regression models to examine candidate predictors of PASS and those of PASS-associated mortality. Covariates were considered for multivariate regression models if they were either statistically significant (P < 0.10) or had odds ratios ≥ 1.5 or ≤ 0.66 on univariate analysis. Candidate predictors included age, race, health insurance, level of poverty at area of residence, chronic co-morbid conditions, obesity, smoking, drug and alcohol abuse, pregnancy-associated complications and high-risk factors, and hospital’s teaching status. Because administrative data sets do not provide information on the temporal course of clinical events, we excluded delivery-related procedures (i.e., cesarean section and operative delivery) and specific pregnancy-associated complications or high risk conditions, except eclampsia/preeclampsia, multiparity, multiple pregnancy, artificial reproduction, iron deficiency anemia, and gestational diabetes. We used this approach because interventions such as cesarean section can be both risk factors for infection and resultant sepsis, but also the result of severe sepsis maternal, requiring emergent intervention [33], while complications such as hemorrhage may precede or follow severe sepsis. Multicollinearity was examined using tolerance (1/variation inflation factor), using a cutoff value < 0.4.

Candidate predictors of PASS-associated mortality included, in addition to those outlined as predictors of PASS, pregnancy-associated complications, delivery procedures, type and number of failing organs (examined in separate models), and use of mechanical ventilation, central venous catheterization, or hemodialysis.

Because TIPUDF masks zip code data in patients with diagnoses of an infection with the human immunodeficiency virus (HIV), alcohol or drug abuse, the level of poverty was not included as predictor of PASS and PASS-associated mortality for the whole cohort, but evaluated in separate models of the subset of hospitalizations with zip code data.

Group data are reported as numbers (percentages) for categorical variables and mean (standard deviation (SD)) or median (interquartile range (IQR)) for continuous variables, as appropriate. Distribution of normality was examined by Kolmogorov-Smirnov test. Categorical data were compared by a two-sided X^2^ test. Mann-Whitney U test and *t*-test were used to compare continuous data, as appropriate. Adjusted odds ratios (aOR) and 95% confidence intervals (95% CIs) were calculated. When examining changes of key characteristics at the start vs. end of past decade we have combined 2-year data to enhance precision of comparisons.

Total hospital charges were examined following standardization to 2010 US dollars, using the annual consumer price index [34]. Linear regression of log-transformed hospital charge data was used to examine trends over study years. Negative binomial models were used to examine trends of hospital length of stay.

All statistical analyses were performed using MedCalc version 12.7.0 (MedCalc Software, Ostend, Belgium) and SAS version 9.3 (SAS Institute, Cary, NC, USA). A two-sided P value < 0.05 was considered significant.

## Results

There were 4,060,201 pregnancy-associated hospitalizations and 1,007 PASS hospitalizations, with 5,347,084 TEPs during the 2001 - 2010 period. The characteristics of PASS hospitalizations are detailed in Tables 1 and 2. Delivery hospitalizations accounted for 37.5% of PASS events, with most of the remainder evenly split between antepartum and postpartum hospitalizations. Most PASS hospitalizations involved Hispanic women, with Medicaid being the most common type of health insurance. Chronic co-morbidities were reported in less than one-third (30.8%) of PASS hospitalizations, with obesity noted in 4.6%.

**Table 1:** The Demographic and Chronic Illness Characteristics of Hospitalizations With Pregnancy-Associated Severe Sepsis

Characteristic	n = 1,007
Age (years, n (%))	
< 20	160 (15.9)
20 - 34	689 (68.4)
≥ 35	158 (15.7)
Race, n (%)	
Hispanic	429 (42.6)
White	308 (30.6)
Black	205 (20.4)
Other	63 (6.3)
Missing	2 (0.2)
Health insurance, n (%)	
Private	324 (32.2)
Medicaid	537 (53.3)
Uninsured	94 (9.3)
Other	50 (5.0)
Missing	2 (0.2)
Poverty level ≥ 20%, n (%)^a^	241 (25.7)
Chronic co-morbidities, n (%)^b^	
Any	310 (30.8)
Myocardial infarction	21 (2.1)
Congestive heart failure	89 (8.8)
Peripheral vascular disease	9 (0.9)
Cerebrovascular disease	29 (2.9)
Chronic pulmonary disease	54 (5.4)
Connective tissue disease	16 (1.6)
Peptic ulcer disease	5 (0.5)
Chronic liver disease	89 (8.8)
Diabetes mellitus	39 (3.9)
Chronic kidney disease	34 (3.4)
Malignancy	6 (0.6)
HIV infection^c^	4 (0.4)
Deyo-Charlson score	
Mean (SD)	0.51 (0.96)
Median (IQR)	0 (0 - 1)
Other conditions, n (%)^d^	
Smoking	30 (3.0)
Drug abuse	52 (5.2)
Alcohol abuse	2 (0.2)
Obesity	46 (4.6)
Teaching hospitals	
Number (%) of hospitals	35 (17.9)
Number (%) of hospitalizations	310 (30.8)

^a^Derived for hospitalizations with non-masked zip code (n = 939). ^b^Based on conditions included in the Deyo-Charlson co-morbidity index. ^c^Human immunodeficiency virus. ^d^Co-morbid conditions not included in the Deyo-Charlson index.

**Table 2:** The Categories of Pregnancy-Related Hospitalizations, Obstetric Risk Factors, Sites of Infection, and Reported Microbiology of Hospitalizations With Pregnancy-Associated Severe Sepsis

Characteristic	n = 1,007
Type of pregnancy-related hospitalization, n (%)	
Fetal loss^a^	104 (10.3)/130 (12.9)
Abortion^a^	9 (0.9)/35 (3.5)
Antepartum	247 (24.5)
Delivery	378 (37.5)
Postpartum	243 (24.1)
Obstetric risk factors, n (%)^b^	
Multiple gestation	9 (0.9)
Retained products of conception	31 (3.1)
Prolonged rupture of membranes	11 (1.1)
Preeclampsia/eclampsia	93 (9.2)
Anemia	350 (34.8)
Gestational diabetes	24 (2.4)
Site of infection, n (%)^c^	
Respiratory	250 (24.8)
Urinary	335 (33.3)
Genital	418 (41.5)
Abdominal	98 (9.7)
Device-related	36 (3.6)
Other	50 (5.0)
Microbiology, n (%)	
Gram-positive	118 (11.7)
Gram-negative	184 (18.3)
Anerobes	9 (0.9)
Other bacteria	31 (3.1)
Fungal	13 (1.3)
Not reported	652 (64.7)

^a^There were 26 fetal loss/induced abortion-related hospitalizations whose only pregnancy-related diagnosis was ICD-9-CM code 639.XX, precluding assignment to either group; upper estimates of the number and percent of fetal loss and induced abortion hospitalizations were provided after the slash for each. ^b^Selected conditions acquired or associated with pregnancy, but not included among other listed co-morbidities. ^c^The total reported percentage exceeds 100, as more than one infection site has been reported for some hospitalizations.

**Figure 1:**
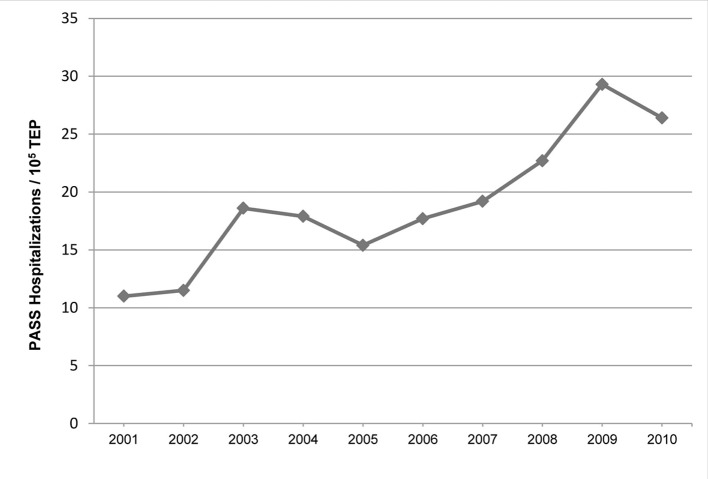
The age-adjusted annual incidence of PASS. PASS: pregnancy-associated severe sepsis; TEP: total estimated pregnancy.

The incidence of PASS increased by 9.7% annually (P = 0.0005) and by 236% from 2001 to 2010 (Fig. 1), rising from 11 to 26 hospitalizations per 10^5^ TEPs. There was no significant change in the annual incidence of PASS on multiple sensitivity analyses assuming a reduced percent of reported hospital discharges in TIPUDF, coupled with assumed higher PASS incidence in non-reporting hospitalizations (P = 0.1311 to P = 0.3843), or by assuming higher rate of fetal loss among pregnant women in the state (P = 0.2454 to P = 0.5063), with findings remaining consistent with our primary analysis.

When we restricted our analysis to PASS events associated with delivery hospitalizations, the incidence of PASS was 10 hospitalizations per 10^5^ live births-years for the whole study period, rising from 6 to 12/10^5^ live births between 2001 and 2010. When classified by other pregnancy outcomes, the estimated incidence of PASS hospitalizations was 1 (5) per 10^5^ induced abortions-years, and 15 (19) per 10^5^ fetal loss-years (using the conservative estimates for abortion and fetal loss to compare incidence of PASS: live births vs. abortion, P < 0.0001; live births vs. fetal loss, P < 0.0001; abortion vs. fetal loss, P < 0.0001).

The genital (41.5%) and urinary (33.3%) tracts were the most common sites of infection. Microbiology data were reported in 35.3% of PASS hospitalizations. Gram-negative bacteria were the most commonly (51.8%) reported microorganisms among PASS hospitalizations with microbiology data.

The key changes of the incidence, clinical characteristics, resource utilization, and outcomes over study period are detailed in Table 3. There was no significant change between 2001 - 2002 and 2009 - 2010 in maternal age ≥ 35 years or frequency of reported co-morbidities. Reported obesity increased from 0.9% to 7.3% of all PASS hospitalizations (P = 0.0211). The most common reported failing organs were respiratory (60%), cardiovascular (48.9%), and renal (25.4%). A single organ failure was reported in 46.4%, and 2 and ≥ 3 organ failures in 27.8% and 25.8%, respectively. The frequency of respiratory failure remained unchanged over study period. However, rates of cardiovascular failure and acute renal failure nearly doubled between 2001 - 2002 and 2009 - 2010, raising from 31.9% to 60.3% (P < 0.0001) and 18% to 31% (P = 0.0099), respectively. At the end of the decade, respiratory failure remained the most common failing organ, followed by cardiovascular failure and acute renal failure. The number of failing organs among PASS hospitalizations rose significantly by the end of last decade, with the rate of ≥ 3 organ failures increased from 8.8% to 35.3% (P < 0.0001). The majority of PASS hospitalizations received care in the ICU, with rate of ICU admission rising from 77.9% to 90% between 2001 - 2002 and 2009 - 2010 (P = 0.0021).

**Table 3:** Changes in the Incidence, Patient Characteristics, Resource Utilization and Outcomes of Hospitalizations With Pregnancy-Associated Severe Sepsis

Variable	2001 - 2002 (n = 113)	2009 - 2010 (n = 300)	P value
Age-adjusted incidence (per 10^5^ TEP^a^)			
All	11	27	< 0.0001
Hispanic	10	27	< 0.0001
White	10	23	< 0.0001
Black	17	37	0.0020
Age ≥ 35 years, n (%)	16 (14.2)	34 (11.3)	0.5381
Chronic co-morbidity, n (%)^b^	32 (28.3)	100 (33.3)	0.3920
Deyo-Charlson score			
Mean (SD)	0.38 (0.81)	0.56 (0.97)	
Median (IQR)	0 (0 - 1)	0 (0 - 1)	0.1173
Obesity, n (%)	1 (0.9)	22 (7.3)	0.0211
Organ failures, n (%)			
Respiratory	65 (57.7)	193 (64.3)	0.2459
Cardiovascular	36 (31.9)	181 (60.3)	< 0.0001
Renal	20 (18)	94 (31)	0.0099
Hepatic	4 (3.5)	22 (7.3)	0.2349
Hematological	28 (24.8)	83 (27.7)	0.6414
Metabolic	14 (12.4)	68 (22.7)	0.0281
Neurological	2 (1.8)	29 (9.3)	0.0152
Number of organ failures			
1	73 (64.6)	106 (35.3)	< 0.0001
2	30 (26.5)	88 (29.3)	0.6626
3	7 (6.2)	59 (19.6)	0.0015
4+	3 (2.7)	47 (15.7)	0.0006
ICU admission, n (%)	88 (77.9)	270 (90)	0.0020
Procedures, n (%)			
Mechanical ventilation	45 (39.8)	138 (46)	0.3099
Central venous catheterization	31 (27.4)	150 (50)	0.0001
Hemodialysis	4 (3.5)	18 (6)	0.4552
Hospital length of stay (days)			
Mean (SD)	14.7 (15.3)	15.7 (21)	
Median (IQR)	9 (6 - 19)	9.5 (5.5 - 18)	0.7939
Hospital charges (dollars)^c^			
Mean (SD)	118,219 (145,240)	191,503 (319,482)	
Median (IQR)	64,034 (34,362 - 150,464)	89,895 (43,614 - 218,357)	0.0141
Disposition, n (%)			
Mortality	11 (9.7)	36 (12)	0.6365
Home	80 (70.8)	191 (63.7)	0.2136
Home care	10 (8.8)	30 (10)	0.8683
Another short-term facility	8 (7.1)	17 (5.7)	0.5613
Long-term care facility	3 (2.7)	14 (4.7)	0.5224
Other^d^	1 (0.9)	10 (1.3)	0.2412
Mortality rate (per 10^5^ TEP)	1.1	3.3	0.0007

^a^TEP: total estimated pregnancy. ^b^Chronic co-morbidities included in the Deyo-Charlson index. ^c^Adjusted for inflation (2,010 dollars). ^d^Inpatient rehabilitation, hospice, leaving against medical advice.

Invasive mechanical ventilation and hemodialysis were required in 41.3% and 5.5% of PASS hospitalizations, respectively, with no significant change over study period (Table 3). Use of mechanical ventilation for ≥ 96 h among PASS hospitalizations requiring mechanical ventilation tended to rise from 48% to 58% (P = 0.3041) over the past decade. Central venous catheterization was increasingly used, rising from 27.4% to 50% of PASS hospitalizations between 2001 - 2002 and 2009 - 2010 (P = 0.0001). There was no significant change in use of the examined interventions among PASS hospitalizations with specific failing organs between 2001 - 2002 and 2009 - 2010: 1) mechanical ventilation: 69.2% vs. 71.5% (P = 0.8486) among those with respiratory failure; 2) central venous catheterization: 44.4% vs. 50.8% (P = 0.6050) among those with cardiovascular failure; 3) hemodialysis: 10% vs. 14% (P = 0.9104) among those with acute renal failure.

Hospital length of stay did not change significantly, with median (IQR) length of stay being 9 (5 - 17.8) days. Inflation-adjusted hospital charges rose by 6.3%/year during study period, increasing from $64,034 to $89,895 between 2001 - 2002 and 2009 - 2010 (P = 0.0141).

Case fatality among PASS hospitalizations did not change significantly over time (P = 0.2079). However, mortality rate rose by 12.4%/year (P = 0.0014), increasing three-fold, from 1.1 to 3.3 per 10^5^ TEPs between 2001 - 2002 and 2009 - 2010 (P = 0.0007).

In sub-analysis, case fatality of PASS hospitalizations over study period was 33.3% (25.7%) for induced abortions, 12.5% (14.6%) for fetal loss, and 13% for live births during delivery hospitalizations. The mortality rates associated with PASS were 0.4 (1.2) per 10^5^ induced abortions, 1.9 (2.8) per 10^5^ fetal losses, and 1.2 per 10^5^ live births among delivery hospitalizations with live births (using the conservative estimates for abortion and fetal loss to compare mortality rates of PASS: live births vs. abortion, P = 0.0531; live births vs. fetal loss, P = 0.1198; abortion vs. fetal loss, P = 0.0063). Among survivors of PASS hospitalization, 74% had routine home discharge.

Candidate predictors of PASS and its associated hospital mortality on logistic regression analyses are detailed in Tables 4 and 5, respectively. The highest risk of developing PASS was associated with chronic liver disease (aOR 41.4) and congestive heart failure (aOR 20.5). In addition, black race (aOR 1.4), obesity (aOR 1.4), poverty (aOR 1.3), lack of health insurance (aOR 1.3), and drug abuse (aOR 3.4) predicted development of PASS. Maternal age ≥ 35 years trended to increase risk of PASS. Among pregnancy-associated complications, preeclampsia/eclampsia was associated with increased risk (aOR 1.3), while gestational diabetes appeared to be protective (aOR 0.5).

**Table 4:** Logistic Regression Analysis of Variables Associated With Development of Pregnancy-Associated Severe Sepsis

Covariate	Adjusted odds ratio (95% CI)^a^	P value
Age ≥ 35 years^b^	1.194 (0.989 - 1.442)	0.0649
Black race^c^	1.354 (1.141 - 1.607)	0.0005
Poverty level > 20%^d^	1.308 (1.125 - 1.520)	0.0005
No health insurance^e^	1.255 (1.010 - 1.558)	0.0403
Smoking	1.050 (0.653 - 1.689)	0.8411
Alcohol	0.631 (0.193 - 2.068)	0.4475
Drug abuse	3.365 (2.462 - 4.600)	< 0.0001
Preeclampsia/eclampsia	1.329 (1.052 - 1.679)	0.0173
Gestational diabetes	0.542 (0.357 - 0.823)	0.0040
Obesity	1.421 (1.011 - 1.996)	0.0431
Iron-deficiency anemia	0.828 (0.578 - 1.187)	0.3036
Chronic co-morbidities^f^		
Myocardial infarction	11.023 (5.932 - 20.484)	< 0.0001
Congestive heart failure	20.485 (15.352 - 27.334)	< 0.0001
Peripheral vascular disease	2.847 (1.212 - 6.692)	0.0164
Cerebrovascular disease	8.624 (4.710 - 15.792)	< 0.0001
Chronic pulmonary disease	1.773 (1.285 - 2.448)	0.0005
Connective tissue disease	2.251 (1.210 - 4.187)	0.0104
Peptic ulcer disease	6.500 (2.000 - 21.130)	0.0019
Chronic liver disease	41.361 (31.531 - 54.266)	< 0.0001
Diabetes	1.771 (1.217 - 2.578)	0.0028
Chronic renal disease	5.581 (3.582 - 9.559)	< 0.0001
Malignancy	4.669 (1.800 - 12.111)	0.0015
HIV infection^g^	4.248 (1.518 - 11.889)	0.0059

^a^95% confidence interval. ^b^Age < 35 years used as referent. ^c^White race used as referent. ^d^Poverty rate ≤ 20% used as referent; modeled only for pregnancy-associated hospitalizations with zip code data (that is, excluding those with a diagnosis of HIV infection, drug or alcohol abuse). ^e^Private insurance used as referent; significant only when modeled without including poverty level (that is, without zip code data, and including pregnancy-associated hospitalizations with a diagnosis of HIV infection, drug or alcohol abuse). ^f^Based on the Deyo-Charlson index. ^g^Human immunodeficiency virus.

**Table 5:** Logistic Regression Analysis of Variables Associated With Hospital Mortality Among Hospitalizations With Pregnancy-Associated Severe Sepsis

Covariate	Adjusted odds ratio (95% CI)^a^	P value
Age ≥ 35 years^b^	1.595 (0.888 - 2.864)	0.1185
No health insurance^c^	2.934 (1.464 - 5.880)	0.0024
Smoking	0.391 (0.088 - 1.742)	0.2512
Drug abuse	3.017 (1.278 - 7.122)	0.0118
Iron-deficiency anemia	1.316 (0.263 - 6.593)	0.7385
Anemia	0.329 (0.183 - 0.593)	0.0002
Preeclampsia/eclampsia	1.063 (0.536 - 2.107)	0.8616
Stillbirth	1.371 (0.407 - 4.622)	0.6105
Operative vaginal delivery	1.589 (0.799 - 3.163)	0.1868
Hemorrhage	1.171 (0.676 - 2.030)	0.5726
Chronic co-morbidities^d^		
Myocardial infarction	1.888 (0.594 - 6.006)	0.2817
Connective tissue disease	2.509 (0.609 - 10.346)	0.2031
Chronic liver disease	1.872 (0.942 - 3.317)	0.0734
Malignancy	7.003 (0.898 - 54.624)	0.0633
Cerebrovascular disease	1.455 (0.483 - 4.379)	0.5047
HIV infection^e^	45.465 (5.180-399.031)	0.0006
Urinary tract infection	0.300 (0.132 - 0.682)	0.0041
Genital tract infection	0.806 (0.427 - 1.522)	0.5056
Organ failures^f^		
Respiratory	2.577 (0.982 - 6.762)	0.0545
Cardiovascular	1.909 (1.167 - 3.122)	0.0100
Renal	1.727 (1.028 - 2.900)	0.0388
Hepatic	1.892 (0.358 - 10.004)	0.4531
Hematological	1.291 (0.731 - 2.278)	0.3791
Metabolic	0.818 (0.459 - 1.457)	0.4951
Neurological	1.637 (0.780 - 3.436)	0.1927
Number of organ failures^f, g^		
2	1.536 (0.766 - 3.076)	0.2265
3	3.827 (1.873 - 7.818)	0.0002
4	2.895 (1.239 - 6.764)	0.0141
Mechanical ventilation	4.540 (2.562 - 8.045)	< 0.0001
Hemodialysis	2.373 (1.126 - 5.000)	0.0231
Central venous catheterization	1.254 (0.781 - 2.014)	0.3496

^a^95% confidence interval. ^b^Age < 35 years used as referent. ^c^Private insurance used as referent. ^d^Based on the Deyo-Charlson index. ^e^Human immunodeficiency virus. ^f^Type and number of failing organs were modeled separately. ^g^One organ failure used as referent.

The highest risk of maternal death was associated with HIV infection (aOR 45.5). In addition, drug abuse (aOR 3.0), lack of health insurance (aOR 2.9), cardiovascular failure (aOR 1.9), increasing number of failing organs (aOR 2.9-3.8), as well as need for mechanical ventilation and hemodialysis, increased the risk of PASS-associated hospital mortality. History of malignancy and chronic liver disease trended to increase odds of death. A diagnosis of anemia (aOR 0.3) and urinary tract infection (aOR 0.3) appeared to have protective impact. Older age, women’s race/ethnicity, and local level of poverty did not appear predictive of PASS-associated hospital mortality. Tolerance values were consistently > 0.4 in predictive models.

## Discussion

We found that the incidence of PASS rose nearly 2.5-fold over the past decade, with the majority of PASS hospitalizations requiring care in the ICU and showing increasing severity of illness and rising resource utilization. The incidence of PASS varied substantially across pregnancy outcomes. The case fatality of PASS, while being relatively low, remained unchanged over the past decade, in contrast with severe sepsis in the general population.

Our study is, to our knowledge, the first population-level examination of the burden of PASS across the full spectrum of pregnancy outcomes and phases of hospitalization, among the at-risk population of TEPs. Our findings document that restriction of the examination of PASS to delivery hospitalizations [22, 24] markedly underestimates the burden of PASS in the obstetric population.

### The epidemiology of PASS

Because we examined the incidence of PASS among the total pregnancy population, our findings are not directly comparable to prior epidemiological studies. Previous investigations reported a wide range of PASS incidence, with varying denominators, commonly restricted to the more readily obtainable live births and fetal deaths. On subgroup analysis restricted to delivery hospitalizations, our findings are comparable to those described by Bauer and colleagues [24], who studied a national administrative data set, using similar ICD-9-based case definition of severe sepsis and reported an incidence of nine PASS hospitalizations per 10^5^ deliveries-years. Our results conflict with the population-based study of live birth hospitalizations in California performed by Acosta et al, reporting 49 PASS hospitalizations per 10^5^ live births-years [22].

In studies based on reports from maternity units [19, 20, 23] or hospitals [21], and including at times an uncertain spectrum of maternal hospitalizations (i.e., lack of reporting on those with fetal loss or postpartum) [19, 20, 23], the incidence of PASS ranged between 21 per 10^5^ delivery-years [20] and 47 per 10^5^ maternities [23] in population-based studies, and from 13 per 10^5^ maternities-years [21] to 35 per 10^5^ delivery-years [19] in local studies. However, the interpretation of prior reports is affected by multiple methodological limitations, including lack of definition of sepsis [20], lack of inclusion of organ failure as prerequisite of severe sepsis [20, 22], reliance on specific “explicit”, but insensitive [35], codes for severe sepsis or septic shock [22], restriction of case definition of severe sepsis to culture-positive patients [19, 21], small sample size [19-21], and use of hospital length of stay [22] or admission to ICU [20, 22] to define severe sepsis. Of note, in a report by Afessa et al [36], severe sepsis was present only in 51% all obstetric patients with sepsis admitted to the ICU, when the authors used consensus definitions [10]. In addition, in a report from the Netherlands, sepsis was not a pre-defined condition for the prospective data collection used in a retrospective review, leading to possible underestimation of PASS events [20].

In the only prospective population-level study to date on PASS, Acosta and colleagues have recently reported on a national cohort of all obstetrician-led maternity units in the UK over 12 months period and including all phases of pregnancy [23]. However, the investigators defined severe sepsis as a suspected or confirmed infection coupled with a modified systemic inflammatory response syndrome, with no requirement of associated organ failure, and with or without need for higher level of care, admission to an ICU or death [23, 37]. Thus, the number of patients with PASS in this cohort remains uncertain, and limits the interpretation of reported findings.

The incidence of severe sepsis associated with induced abortion or with fetal loss has not been previously reported, to our knowledge. Our findings underscore the remarkable safety of contemporary legal abortion practices, while it appears that fetal loss is associated with a higher incidence of severe sepsis than that found among delivery hospitalizations. Further studies are required to corroborate these findings and determine the sources of the observed differences.

The finding of progressively rising incidence of PASS in the present cohort extends the near-identical results by Bauer and colleagues among delivery hospitalizations, who reported a rise in the incidence of PASS by 10%/year [24]. Several possible explanations may be considered for the apparent rise of incidence of PASS in our cohort. Our findings may simply reflect increasing coding of organ failure among septic patients without PASS. However, this explanation is not supported by the concurrent increased use of lifesupport-related interventions among respective organ failures, rising rate of ICU admission, increased hospital charges, and lack of corresponding expected decrease in case fatality.

Increased clinician awareness of a specific clinical condition and thus increased documentation should be considered as an alternative source of an apparent rise in its incidence. However, severe sepsis remains a rare complication of pregnancy, and most clinicians in the state would not have encountered on average a single patient with PASS in a given year. Moreover, the limited clinician awareness of PASS has been underscored by multiple reports of prevalent lack of timely recognition and care of women with this complication [20, 38, 39]. Of note, although there has been likely marked increase in clinicians’ awareness of severe sepsis in the general population, a recent report by Rohde and colleagues noted that severe sepsis was documented by treating clinicians only in 47% [40].

A recent study from the UK noted the rising mortality rate associated maternal sepsis, based on chart reviews [38]. As there have been no reports of rising case fatality associated with maternal sepsis, it is plausible that the rise in mortality rate in the UK reflects actual increasing incidence of PASS and may explain our observations. Nevertheless, despite the aforementioned considerations, the use of administrative data cannot allow definitive distinction between increasing documentation of PASS events and true increase of PASS incidence in the present cohort or the report by Bauer et al [24].

If the incidence of PASS is indeed rising, the sources driving this change remain unclear. Several investigators have noted the rising incidence of conditions and procedures leading to maternal severe sepsis and septic shock, including rising maternal age, obesity, chronic illness, use of cesarean section, and use of invasive procedures [18]. While the aforementioned factors are well associated with risk of infection, their role in progression from infection to severe sepsis among obstetric patients has not been systematically examined. In addition, increasing virulence of infecting microorganisms and increasing resistance to antimicrobials may have contributed to the observed incidence changes. We found no significant change in PASS hospitalizations older than 35 years or reports of chronic illness. Obesity was increasingly noted in our cohort, though its rate was markedly lower than that reported in the obstetric population [41], reflecting underreporting in administrative data sets [42]. The use of administrative data precluded examination of evolving patterns of use of invasive procedures that preceded PASS, rather than performed afterwards, or examination of evolving microbial virulence or resistance patterns.

The available contemporary epidemiological reports on PASS have been restricted to Western Europe and the US. However, as noted earlier, the bulk of the global burden of maternal sepsis and thus of PASS is affecting disproportionately developing countries. Thus, data from developing countries (and other regions) are urgently needed to better understand the current epidemiology and the public health impact of PASS in these areas. However, these types of investigations can be challenging, especially in resource-limited areas, often lacking sufficient local epidemiological expertise and consistent ability by the relatively limited number of clinicians to accurately diagnose and report these complications.

### Clinical features of PASS

PASS was most commonly reported during delivery hospitalizations in the present cohort, followed by PASS events during the antepartum and postpartum hospitalizations. On the other hand, PASS was reported most commonly during the postpartum period in other population-based studies [20, 23], though these findings may have been affected by the noted limitations of case definition of severe sepsis. PASS related to abortion was reported in 6% [43] to 7% [39] in local studies. It is unclear whether some of the studies based on reports from maternity units included postpartum hospitalizations [20, 23] or PASS events associated with induced abortion or miscarriage [19, 23]. The administrative data used in the present study precluded separation of antepartum vs. postpartum PASS events during delivery hospitalizations. However, our results demonstrate that the majority of PASS events encountered by clinicians do not occur during delivery hospitalizations.

Hispanic women were the largest ethnic group in our cohort and that reported by Acosta et al on the California population [22], while constituting 17% in the report by Bauer et al [24], reflecting varying state vs. national demographics. The rate of uninsured PASS hospitalizations was more than 2.5-fold higher in our cohort that in the national population reported by Bauer et al [24], in line with the markedly higher uninsured population in Texas.

Chronic co-morbidities were reported in less than one-third of PASS hospitalizations, reflecting the generally healthy obstetric population, with congestive heart failure and chronic liver disease being the most commonly reported conditions. Previous population-based studies of PASS varied in the detail of examined co-morbidity burden of PASS patients. Acosta et al documented only occurrence of diabetes and chronic hypertension among live birth PASS hospitalizations [22], while Bauer et al reported a broader, but still selective range of chronic co-morbidities, with the most common being congestive heart failure, systemic lupus, and chronic liver disease [24]. However, neither of these studies provided data on the overall frequency of any chronic co-morbidity (of those examined) among PASS hospitalizations. Our findings are similar to those reported by Zwart and colleagues, who found one or more chronic co-morbidities in 28% among obstetric patients admitted to the ICU in the Netherlands [44]. There was no significant change in the frequency of chronic co-morbidity among PASS hospitalizations by the end of the last decade. The trends of chronic co-morbidity burden were not reported in other studies of PASS.

The genital and urinary tract infections were the most commonly reported sites, similar to other studies on PASS [20, 22, 24] and in line with infections in the obstetric population. Respiratory tract infections were reported in one in four PASS hospitalizations. Our findings are comparable to the reported respiratory infections in 30% PASS hospitalizations in the study by Bauer et al [24], while contrasting the occurrence of these infections only in 5.5% of the patients studied by Acosta et al [23]. The sources of the rare occurrence of respiratory tract infections in the latter study are unclear, but may be related in part to the noted methodological differences in case definition.

Patient-level data on the pathogens associated with PASS are limited due to the rarity of this complication in the obstetric population. Most of the available data are derived from that on the microbiology among infected obstetric patients who are not necessarily severely septic. It is presently unknown to what extent these data apply to PASS population. We found predominance of Gram-negative bacteria among reported microbiology data. Other studies of PASS found either equal rates of Gram-positive and Gram-negative bacteria [24] or predominance of Gram-positive bacteria [23]. Antimicrobial resistance patterns were not reported in the prospective study by Acosta et al [23]. Microbiology data were reported only in a minority of PASS hospitalizations in our cohort and those reported by Bauer et al [24], reflecting a common constraint of administrative data sets [45], and limiting the generalizability of our findings.

The respiratory, cardiovascular, and renal systems were the most commonly reported organ failures in our cohort. Development of organ failure was examined inconsistently in most studies of PASS. Respiratory failure was the most commonly affected system among PASS patients, reported in 44% [43] to 70% [39] in local studies, and 34% in a population study by Bauer and colleagues [24]. Renal failure was reported between 16% [24] and 37% [39]. Acosta et al did not describe systematically the occurrence of failing organs in their population [22]. Hematological dysfunction was especially common in local studies, ranging between 39% [43] and 43% [39] of patients, while reported in 19% of PASS hospitalizations in a population-based study [24]. Neurological dysfunction appears uncommon, described in 8% [24] of hospitalizations to 11% [43] of patients. Our findings conflict with the markedly lower rates of individual organ failures reported by Bauer et al [24], though the investigators used similar or, at times, broader ICD-9-based definitions. The sources of the differences with the latter study are uncertain, though they may be the result of different population mix, as reflected by varying outcomes of sepsis across states in the general population [46]. Only one previous study of PASS, a two-hospital cohort, reported by Snyder and colleagues, described the distribution of the number of failing organs in PASS, with single organ failure in 40%, with 2 and ≥ 3 in 27% and 33% of their patients, respectively [39], similar to our findings. Our findings are comparable to those reported in the general population with severe sepsis [1, 2].

The rates of most reported organ failures and the number of failing organs rose substantially over the past decade. The changes were most dramatic among PASS hospitalizations with cardiovascular failure and those ≥ 3 organ failures, rising nearly two-fold, and four-fold, respectively. The increased rates of organ failure and the number of affected organs may reflect over-coding. However, as noted earlier, this explanation is not supported by the concurrent rise in use of examined organ-specific lifesupport-related interventions, rising rates of ICU admission, and increased hospital charges. Nevertheless, the factors driving the observed changes in organ failures type and number in our cohort are unclear, as there was no increase in aging or substantial rise on chronic co-morbidities over the past decade. Changes in the virulence and antimicrobial resistance of infecting pathogens may have contributed to the increased severity of illness among PASS hospitalizations. However, these factors readily cannot be adequately examined in administrative data sets.

### Resource utilization

The majority of PASS hospitalizations were admitted to ICU, reaching an admission rate of 90% by the end of the last decade. Our findings are comparable to the 79% ICU admission rate reported by Kramer and colleagues [20], though are markedly higher than the 31.2% admission rate noted by Acosta et al [23]. However, it is unclear how many patients had severe sepsis in the latter cohorts. ICU admission rates were not reported in other studies of PASS. Although multiple population-level studies examined ICU utilization among obstetric patients [44, 47], none focused specifically on severe sepsis. The rate of ICU admission in our study is markedly higher than that reported in the general population with severe sepsis [48, 49]. It can be postulated that clinicians in the state may have had a lower threshold for ICU admission among pregnant patients with severe sepsis. However, in a preliminary report comparing PASS hospitalizations with age-similar, non-pregnant women with severe sepsis, we found similar high rates of ICU admission (85% vs. 83%, respectively) [50]. Nevertheless, ICU utilization patterns can vary nationally [51] and regionally [52].

Mechanical ventilation and central venous catheterization were commonly used among PASS hospitalizations, with infrequent need for hemodialysis. Use of lifesupport-related interventions was described infrequently in prior studies of PASS. Need for mechanical ventilation for ≥ 96 h did not change significantly by the end of the last decade, contrasting the reported marked decline in the cohort described by Bauer et al [24]. The sources of the difference are unclear, especially in the absence of data on the trends of respiratory failure among PASS hospitalizations in their study. Acosta and colleagues reported use of “ventilation” in 7.6% of their patients without septic shock [22]. However, the investigators did not define ventilation (i.e., invasive vs. non-invasive) and the markedly low rate of its use was likely affected by the overly broad definition of severe sepsis. Reported hemodialysis use ranged from about 5% [24] of PASS hospitalizations in a national population study to 10% in a local cohort [39]. Further studies are required on the use of lifesupport-related interventions in patients developing PASS.

PASS events required prolonged hospitalization. Data on hospital length of stay varied in prior studies of PASS, ranging from 10 to 19 days [20] in a population-based study in the Netherlands and averaged 15.1 days among survivors of septic shock in a small case series in the US [43]. The median hospital stay was 5 days among hospitalizations with severe sepsis without shock in the report by Acosta et al [22]. However, the latter figure likely stems from the overly broad definition of severe sepsis in that study. Our findings are similar to those reported in the general population with severe sepsis [2], attesting to the severity of illness in the present cohort, despite being younger and healthier than the former.

The fiscal burden of PASS hospitalizations has not been previously examined. We found that the total hospital charges rose substantially during the past decade. The mean hospital charges for PASS hospitalizations in 2009 were 10-fold higher than the corresponding hospital charges for pregnancy-associated hospitalizations in the state [53], underscoring the high morbidity of PASS patients.

### Outcomes of PASS

The case fatality associated with PASS hospitalizations was relatively low compared with severe sepsis in the general population [1, 2]. The case fatality of PASS was not reported by Bauer et al [24], and ranged from 0.8% in patients without septic shock [22] and 1.4% [23] through 7.7% [20] in population-based studies. The very low hospital mortality in the former two studies suggests that a substantial number of their patients may not have had severe sepsis. Local studies reported 10% case fatality of PASS [39] and ranging from 28% [43] to 33% [39] for septic shock. Of note, while not focusing specifically on severe sepsis, Zwart and colleagues reported case fatality of 9.1% among obstetric patients with sepsis who were admitted to ICU in the Netherlands [44].

It has been suggested by several investigators [12, 18] that the low case fatality of maternal sepsis is due to patients’ younger age and generally better baseline health status. However, there were no reports, to our knowledge, of direct comparison of patients with PASS and age-similar, non-pregnant women. In a recent preliminary report we found lower case fatality among PASS hospitalizations aged 20 - 34 years when compared with contemporaneous age-similar, non-pregnant women with severe sepsis, without reported chronic co-morbidities (6.7% vs. 14.1%, respectively) [50]. Further studies are needed to corroborate these findings and to provide better insight into the comparative response to infection among pregnant versus non-pregnant severely septic women.

Although PASS associated with induced abortion was extremely rare, it had the highest associated case fatality, while case fatality among PASS hospitalizations associated with fetal loss was comparable to that found among those during delivery hospitalizations. The rarity of severe sepsis associated with legal abortion and our study design further limit analysis of possible sources for the differences in case fatality between the examined groups. Our findings of the mortality rates among PASS hospitalizations associated with induced abortion and fetal loss can be best put in perspective when considering the study of Grimes and colleagues on fatal septic abortion in the US. The investigators found that during the period of 1975 - 1977, the mortality rates were 0.4 and 0.6 per 100,000 legal abortions and fetal loss, respectively [54]. It is sobering to consider that even when using conservative estimates, the contemporary mortality rate of severe sepsis associated with induced abortion remained virtually unchanged over the past four decades, while that associated with fetal loss appears three-fold higher, despite the major transformation in clinical practice over this period.

A key finding of the present study has been the unchanged case fatality of PASS hospitalizations over the past decade. This finding may be considered to represent improved patient care among patients with increasing severity of illness. However, our results contrast the consistently reported progressive decline in case fatality of severe sepsis in the general population [1, 2, 55], despite the noted rise in the number of failing organs and population aging. The decreasing case fatality in the general population with severe sepsis has been attributed in part to increased clinician awareness and improved patient care [1]. However, there is no evidence of improved clinician awareness of severe sepsis associated with pregnancy or its improved care over time and, as demonstrated by several investigators, patient care is often inadequate among fatalities associated with maternal sepsis. As demonstrated in our study, PASS remains an uncommon complication in the obstetric population and most physicians or hospitals may not encountered a patient with PASS in any given year. The initial manifestations of severe sepsis in obstetric patients may overlap with those of pregnancy [18] and the site of infection may not be readily apparent in many patients [43]. As a result, clinical diagnosis of PASS can be uniquely challenging, and patients’ clinical course can become rapidly fatal. Kramer and colleagues reported that the time from onset of infection to death was less than 24 h in 50% of the patients who died due to severe sepsis [20]. Similarly Snyder et al noted that there was rapid clinical deterioration among all PASS patients who died [39]. Lack of adequate care among fatalities associated with PASS appears prevalent. Kramer and colleagues have found that among women who died due to severe sepsis, a substandard care analysis showed delays in diagnosis and/or therapy in 38% of patients [20]. In the report of the confidential enquiry on maternal deaths in the UK, Cantwell and colleagues reported that “substandard care” occurred in 69% of those who died due to sepsis [38]. The authors recommended “going back to the basics”, including among other recommendations, mandatory, audited training of all clinical staff in the identification and initial management of pregnancy-associated sepsis [38]. There has not been comparable systematic evaluation of care of PASS in the US. Because maternal death due to sepsis appears largely preventable [16, 38], while its mortality rate is rising [38], a workable systematic approach to foster timely recognition and care of severe sepsis in obstetric patients remains urgently needed.

Most hospital survivors of PASS in the present cohort were discharged home. The disposition of hospital survivors has not been examined in previous population studies of PASS. Our findings contrast those among survivors of severe sepsis in the general population, with home discharge rates only about half as those of PASS [1]. The observed difference in disposition among survivors of PASS vs. severe sepsis in the general population is likely in part due to the younger age, markedly lower burden of chronic illness, and possibly predominant infections of the genitourinary tracts which may be more readily controlled. Although the distribution of the number of failing organs was comparable to that in the general population with severe sepsis, the severity of individual failing organs among PASS hospitalizations could not be determined from administrative data, but may have been lower than comparable organ failures in the non-obstetric severe sepsis population.

Severe sepsis can be associated with multiple long-term sequelae among survivors, including higher long-term mortality than that of the general population, lingering cognitive and physical dysfunction, as well as mental health sequelae, including depression, anxiety, and post-traumatic stress disorder [56, 57]. There are currently no reports on the long-term impact of PASS. Further studies are urgently needed to better understand the post-hospitalization outcomes of survivors of maternal severe sepsis, to better address prevention and need for long-term care interventions.

### Predictors of PASS and its associated mortality

Many of the risk factors for development of PASS were similar to those reported by other investigators, including chronic illness [22, 24], lack of health insurance [22], preeclampsia/eclampsia [19, 22], and poverty [20]. The risk of PASS was especially high among women with congestive heart failure and chronic liver disease, similar to the findings by Bauer et al [24]. As noted, we could not examine the role of operative procedures or specific obstetric complications such as hemorrhage, due to our use of administrative data. For example, cesarean section can be a risk factor for sepsis, but can also follow septic events [33]. However, induced labor [20, 21], cesarean section [19-21], and premature rupture of membranes [20] were found to be predictors of PASS in studies based on chart review. Obesity, a well-known risk factor for infections in pregnancy [58], has been associated with increased risk of PASS in our cohort, in contrast to the study by Bauer et al [24]. However, obesity has been reported in more than a quarter of pregnant women [41] and it is likely that the rate of obesity was underreported in our population and especially in Bauer’s cohort, as can be the case in administrative data sets [42]. The impact of drug abuse, associated with more than three-fold higher odds of PASS in our cohort, has not been examined in prior studies. Older maternal age was not a significant predictor of PASS in our cohort, contrasting other reports [20, 24]. However, we controlled our predictive models for broader array of potential confounders, including organ failure, which may have affected our findings. We found an unexpected “protective” impact of gestational diabetes on development of PASS. Our study design precludes inferences into the mechanisms underlying associations. However, it may be postulated that women diagnosed with gestational diabetes could have had increased monitoring and possibly different care than other obstetric patients. Nevertheless, due to the observational, retrospective design of our study, we cannot exclude an effect of residual confounding. Our findings provide assessment of risk attributes that are either potentially modifiable or identify a patient subset requiring especially heightened clinician vigilance.

Our study is the first to examine predictors of mortality associated with PASS. Although reported only in a minority of PASS hospitalizations, the risk of maternal death was especially high among those with HIV infection, and trended to increase among those with history of malignancy and chronic liver disease. In addition, drug abuse, lack of health insurance, development of selected organ failures, rising number of failing organs and need for mechanical ventilation or hemodialysis increased, as expected, the odds of death. Of note, our findings complement the adverse prognostic impact of lack of health insurance in severely septic patients in the general population [59]. On the other hand, urinary tract infection was associated with reduced risk of death among PASS hospitalizations, similar to reports in the general population [60]. A diagnosis of anemia was associated, unexpectedly, with reduced odds of hospital death. The pathobiology underlying this association among PASS hospitalizations is uncertain and, as noted earlier, we cannot exclude an effect of residual confounding, especially with reported increased risk of severe sepsis among anemic women [21]. Of note, increased maternal age, minority race, or poverty did not affect maternal risk of death, once controlled for other confounders. Further studies are warranted to corroborate our findings.

Our findings should be considered in the context of several limitations. First, a retrospective design and use of an administrative data set with their attendant limitations affects the interpretation of our results. However, the rarity of PASS can be a challenge for alternative population-level approaches to study this condition. In addition, the de-identified data do not allow accounting for multiple hospitalizations by the same patient during specific period. However, similar approach with the aforementioned limitations was used by other investigators [24].

The optimal ICD code-based approach to identify patients with severe sepsis in administrative data sets remains unsettled. A recent study by Gaieski and colleagues, using a national data set demonstrated nearly 3.5-fold difference in the number of identified severe sepsis hospitalizations, and marked variability in the number of failing organs and case fatality between four different code-based methods, although all trended comparably over time [5]. We chose a conservative approach to identify PASS hospitalizations, in part because it produced a distribution of the number of failing organs and estimates of disease burden comparable to epidemiological chart-based studies of severe sepsis [61, 62], and we observed comparable findings in our cohort. As noted earlier, our findings of the distribution of the number of failing organs was similar to those of a chart-based study of PASS [39], supporting our case identification approach. Nevertheless, we cannot exclude a possibility of underestimating occurrence of PASS hospitalizations.

Prenatal care may have affected the risk of infection and resultant development, clinical course, and outcomes of PASS. Because we used de-identified data, occurrence of prenatal care and its adequacy across pregnancy-associated hospitalizations could not be examined and may have affected the clinical findings, resource utilization and results of predictive models. Our findings of the adverse impact of lack of maternal health insurance on the development of PASS and its associated mortality underscore the positive preventive impact of proper perinatal care.

Pregnancies ending in fetal loss are not readily tracked across populations, and our denominator data for incidence calculations reflect estimates. However, our methodology for estimating the fetal loss population was based on a population-linked study geared specifically to avoid inflating fetal loss rates through inclusion of events occurring prior to planned abortion, and our resulting incidence estimates of derived TEP remained robust on sensitivity analyses, including a fetal loss rate exceeding prior reports. In addition, because we used an administrative data set, we cannot exclude the possibility that fetal loss followed, rather than preceded PASS in some hospitalizations. Thus, our estimates of PASS incidence associated with fetal loss should be considered to represent either of these sequences. However, the morbidity and mortality impact noted in this patient group remains unchanged regardless of the sequence of preceding events. Finally, given the very small number of PASS events associated with fetal loss, alternative study methods of its epidemiology may not be practical.

Because administrative data sets preclude use of established severity-of-illness scores, we used the number of failing organs as a surrogate measure. However, similar approach was employed by other investigators [1, 28], and the number of failing organs was associated with incremental risk of death among PASS hospitalizations.

We examined PASS in a large state with diverse population. However, the characteristics of PASS and the required resources for PASS patients may vary across states and nationally. Further studies on PASS are needed in other populations in both developed and developing countries.

The use of administrative data in our study precluded access to information on the timeliness of diagnosis and care processes of PASS, which may have varied across institutions and individual clinicians and may have affected the observed resource utilization and outcomes. However, similar constraints affect interpretation of prior population-level studies of PASS [22, 24] and severe sepsis in the general population [1, 2, 48]. Because the state of Texas does not provide tools to convert hospital charges to costs, we reported hospital charges rather than costs of care, limiting comparisons with other cost data. However, the available charge data allowed comparisons within state population.

Zip code masking among hospitalizations with diagnoses of HIV infection, and drug or ethanol abuse restricted the assessment of the prognostic role of local economic state in this subgroup. However, the aforementioned diagnoses accounted for less than 7% of our cohort. Finally, although we have performed extensive adjustment for confounders in our predictive models, we cannot exclude residual, unaccounted confounding that may have affected our results.

### Conclusions

We have reported the largest study to date on PASS across the spectrum of pregnancy outcomes. Our study is the first to examine evolving changes in chronic illness, ICU admission, organ failure, resource utilization and outcomes of PASS patients, aiming to align population-level study of PASS with contemporary investigations of severe sepsis in the general population.

Although PASS remains uncommon, its incidence appears to be rapidly rising, with associated increased severity of illness, prevalent admission to ICU, and rising resource utilization and mortality rate. The case fatality associated with PASS remained unchanged over the past decade, in contrast with the consistent decreasing case fatality among patients with severe sepsis in the general population, highlighting the urgent need to increase clinicians’ awareness and improve care of affected obstetric patients. Although severe sepsis was very rare among women undergoing induced abortion, the associated case fatality was the highest compared to other PASS patients, and its mortality rate has not changed over the past four decades. PASS is more likely to develop among minority women and those with chronic illness. Pregnant women with history of drug abuse and lacking health insurance are at high risk of both developing and dying with PASS, requiring extra vigilance for early diagnosis and targeted intervention. Further studies are needed to better understand the burden of PASS across the spectrum of pregnancy outcomes, in both developed and developing countries, to identify the best practices to improve systemic approach to assure effective care, and to provide insight into its long-term sequelae.
